# Suppressing the *Neurospora crassa* circadian clock while maintaining light responsiveness in continuous stirred tank reactors

**DOI:** 10.1038/srep10691

**Published:** 2015-06-02

**Authors:** Allison L. Cockrell, Russell K. Pirlo, David M. Babson, Kathleen D. Cusick, Carissa M. Soto, Emily R. Petersen, Miah J. Davis, Christian I. Hong, Kwangwon Lee, Lisa A. Fitzgerald, Justin C. Biffinger

**Affiliations:** 1 Chemistry Division, US Naval Research Laboratory, 4555 Overlook Ave., SW., Washington, DC, 20375, USA; 2 Center for Bio/Molecular Science and Engineering, US Naval Research Laboratory, 4555 Overlook Ave., SW., Washington, DC, 20375, USA; 3 Nova Research Inc., 1900 Elkin St., Suite 230, Alexandria, VA, 22308, USA; 4 Howard University, Washington, DC. 20057; 5 Department of Molecular and Cellular Physiology, University of Cincinnati, Cincinnati, OH 45267, USA; 6 Department of Biology, Rutgers University, Camden, NJ, 08102, USA

## Abstract

*Neurospora crassa* has been utilized as a model organism for studying biological, regulatory, and circadian rhythms for over 50 years. These circadian cycles are driven at the molecular level by gene transcription events to prepare for environmental changes. *N. crassa* is typically found on woody biomass and is commonly studied on agar-containing medium which mimics its natural environment. We report a novel method for disrupting circadian gene transcription while maintaining light responsiveness in *N. crassa* when held in a steady metabolic state using bioreactors. The arrhythmic transcription of core circadian genes and downstream clock-controlled genes was observed in constant darkness (DD) as determined by reverse transcription-quantitative PCR (RT-qPCR). Nearly all core circadian clock genes were up-regulated upon exposure to light during 11hr light/dark cycle experiments under identical conditions. Our results demonstrate that the natural timing of the robust circadian clock in *N. crassa* can be disrupted in the dark when maintained in a consistent metabolic state. Thus, these data lead to a path for the production of industrial scale enzymes in the model system, *N. crassa*, by removing the endogenous negative feedback regulation by the circadian oscillator.

Living organisms have developed complex regulatory rhythms that allow for adapting to environmental cues such as light, temperature, and carbon source availability based on a 22-23.5 hr cycle. Many cellular processes (including the cell cycle) are linked to a light-responsive circadian clock, which provides a reference point for metabolic cycles^1^,^2^,^3^,^4^. This biological clock provides a sense of timing, so that molecular mechanisms are synchronized to maintain necessary cellular functions^5^. Altering these rhythms can have deleterious effects on an organism. For instance, patients with dysfunctional circadian gene regulation have been linked to increased risks of developing cancer, diabetes, and psychiatric and/or mood disorders^6^,^7^.

Circadian rhythms are derived from rhythmic gene transcription that is regulated by a series of positive and negative feedback loops^8^. This regulatory cycle is conserved across many species from humans to fungi^9^. The filamentous fungus *Neurospora crassa* is considered a model organism for studying circadian gene oscillations. One of the core clock components in this fungus is the *frequency* gene (*frq*)^10^. The expression of *frq* is activated by a heterodimer complex known as the “White Collar Complex”, or WCC. This complex is comprised of two PAS-domain (*Drosophila* period clock, aryl hydrocarbon receptor, single-minded protein) containing clock proteins, White Collar-1 (WC-1) and White Collar-2 (WC-2)^11^,^12^,^13^. During the circadian late night and early morning, *frq* transcription is promoted by the WCC, with FRQ concentrations peaking by the circadian mid-day^4^. FRQ participates in a negative feedback loop to inhibit *frq* gene transcription^14^. Due to this inhibition, *frq* transcript levels decline in the circadian afternoon/early evening. Additionally, FRQ acts to up-regulate WC-1 translation and *wc-2* transcription^14^,^15^. The vivid (*vvd*) gene has also been shown to be under WCC control and is involved in photoadaptation in *N. crassa*^16^. VVD competitively interacts with WC-1 to inhibit WCC formation (and thereby the transcription of *vvd* as well)^17^. These interlocked positive and negative feedback loops are important to preserve robust circadian rhythms in a living organism^14^,^18^.

Traditionally, the circadian clock in *N. crassa* has been studied on agar-based culture media within glass tubes (termed “racetubes”) (Fig. 1), petri dishes^19^,^20^,^21^, or more recently in microfluidic polydimethylsiloxane (PDMS) frames with precise gas control^22^. The period of the circadian clock can be determined from the simultaneous formation of rhythmic conidial “bands” (the physical manifestation of the asexual sporulation state in *N. crassa*) in conjunction with the development of branching hyphae from the initial inoculation point. The design of these assays are ideal to simulate how *N. crassa* survives in nature where the results of cellular metabolism (for example: changes in local pH, nutrient availability and waste accumulation) are not controlled but instead are responded to by the metabolism of the fungal cells. However, the results from cellular growth on uncontrolled solid substrate assays, such as those described above, lead to slow diffusion of metabolic by-products, formation of dense fungal mats, and limited nutrient availability which ultimately leads to significant localized physiological changes^23^,^24^.

In addition to observing circadian rhythms in racetubes, these cycles are also seen in batch submerged cultures^25^. In batch culture experiments, a mycelium disc is transferred to a nutrient-deficient medium and the cells are harvested periodically to analyze gene transcription and protein expression levels. While these assays are designed for understanding the fungus in a liquid culture, some factors such as nutrient diffusion, localized pH gradients, and oxygen tension, are not well-controlled and the resulting environmental stressors could impact the circadian clock independently. These stressors should be controlled in order to determine if they are affecting the physiological state of the fungus in a way that influences the circadian clock^26^. This can be accomplished by cultivating *N. crassa* within bioreactors which allows for the constant removal of medium, cells, and waste material with the input of fresh medium/nutrients (termed “continuous culture”)^27^ which minimizes potential changes in the culture medium. An image and schematic of the reactor used in these experiments is shown in Fig. 1. In addition to the continuous flow of medium in and out of the reactor, several key environmental growth variables (dissolved oxygen, pH, and temperature) are controlled (Supplementary Fig. S1).

In the studies presented here, cultures of *N. crassa* were maintained in continuous stirred tank reactors (CSTRs). The expression of four core *N. crassa* clock component genes *frq, wc-1, wc-2*, and *vvd* were analyzed with RT-qPCR to determine how the circadian clock was influenced during CSTR cultivation. Light responsiveness was also investigated by growing cultures in either constant darkness (DD), or with 11 hr periods of alternating light or dark exposure (LD). Our results demonstrate that circadian gene regulation was suppressed in DD cultures while genes linked to the circadian clock were up regulated during light-on cycles in LD experiments. To understand how downstream genes responded to DD and LD conditions, the expression of three clock-controlled genes (*ccg*), *ccg*-1, *ccg*-4, and *ccg*-14, were also analyzed. These genes displayed arrhythmic expression patterns under DD conditions and were up regulated with light exposure.

## Results

### Culture and operation of CSTR containing *N. crassa*

A *N. crassa* strain with *ras-1*^*bd*^ background^15^,^28^ was used for these experiments with 1.0% glucose (w/v) as the sole carbon source. Two different light exposure cycles (all-dark (DD) or 11 hr light-dark (LD) cycles) were used to determine the impact of light on circadian gene transcription at steady state in a CSTR. The production of CO_2_ served as the metric by which steady state was defined. Both LD and DD experiments consistently produced 3500–4000 ppm CO_2_ throughout the duration of the experiment (Fig. 2). For DD, once the culture reached a steady metabolic state the reactor was exposed to light for 24 hrs and then kept in constant darkness (maintained with photographic darkroom safelights) for the remainder of the experiment. For LD experiments, after the initial 24 hr light exposure the cultures were exposed to 11 hr light/dark cycles during steady state starting with a dark cycle. The dilution rate of the culture medium and waste removal to maintain steady state was 1.0 ± 0.1 mL/min.

**Figure 1:**
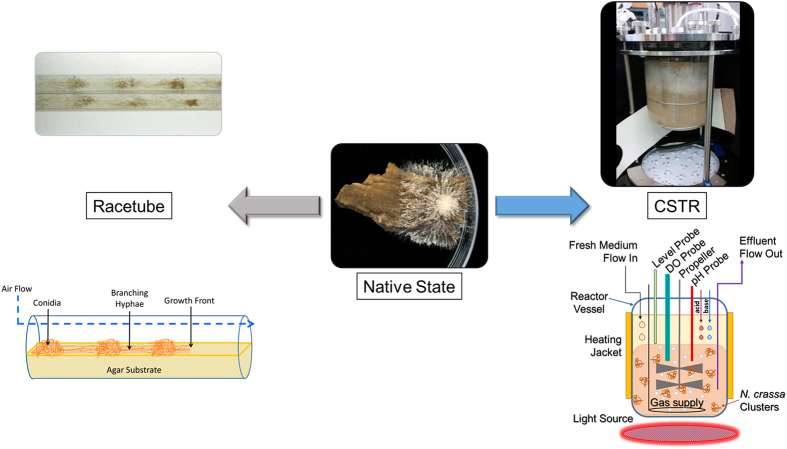
Pictorial and schematic comparison between growth of *N. crassa* in racetubes, unaltered, and within CSTRs with schematic details.

The growth of a filamentous fungus while achieving a stable metabolic environment has been shown to be difficult^29^ since submerged fungal cultures, such as *N. crassa,* form “mats” comprised of densely packed hyphae^30^ These fungal mats accumulate in and around all CSTR components (Fig. 3a). The addition of polyacrylic acid (Junlon, average MW ~100,000) to the culture medium^26^,^31^ and a second bioengineering technique termed “dynamic agitation” were required to disperse the fungal mats that formed in the reactor. The dynamic agitation method cycled the propeller periodically from 400 rpm to a high (1000 rpm) and a low (100 rpm) revolution rate (Fig. 3b) while continuously removing the freely dispersed cells with independent peristaltic pumps. The cell morphology (hyphae and conidial states) from CSTRs and batch cultures were indistinguishable determined by fluorescence microscopy (Supplementary Fig. S3). The combination of Junlon and dynamic agitation produced a uniform distribution of cells (Fig. 1) capable of being removed from the vessel and allowing for a steady metabolic state to be established.

Racetube experiments were performed to confirm that the cultures used to inoculate the CSTRs were capable of producing the expected circadian rhythm using a standard assay and in the presence of all the media components used in the CSTRs (Fig. 1). Reproducible 23.4 ± 0.5 hr conidial banding patterns were observed from these control racetube experiments and the conditions are described in the methods section. The results from the racetubes confirm the expected period of conidial banding using these culture conditions prior to inoculation into the CSTRs.

### Circadian gene transcription at steady state in constant darkness (DD)

Periodic patterns in *frq* transcription are considered a reliable indicator of circadian gene oscillations in *N. crassa*^9^. These patterns were determined by RT-qPCR using stable reference genes for continuous culture conditions (*btl* and *vma2*)^32^. The periodicity of this overt rhythm can be entrained by exposing the cultures to light. When *N. crassa* was cultivated under controlled DD conditions, the transcription levels of *frq* declined immediately after the light was turned off and remained low and arrhythmic for the remainder of the experiment (Fig. 4a). Additionally, low-amplitude and arrhythmic expression patterns were observed with *wc-1, wc-2*, and *vvd* in the DD experiments (Fig. 4b–d). The absence of a rhythm in these gene transcripts was also confirmed using an SAS/ETS (www.SAS.com) time series analysis method (Supplementary Fig. S5-S7), in which the time-dependent expression data from three biological replicates were analyzed for periodic trends and cyclic patterns.

In addition to these key circadian genes, there are many “clock-controlled” genes (*ccg*s) that reside in physiological output pathways^29^ and result in higher order control over the *N. crassa* transcriptome. Three of these *ccg*s (*ccg-1, ccg-4,* and *ccg*-*14*) were chosen based on their established role in circadian cycles in *N. crassa*^26^,^33^,^34^. Under our DD culture conditions there were no rhythmic fluctuations in *ccg* expression (Fig. 4e–g) indicating that these *ccgs* were not expressed with defined periods and amplitudes as would be expected if controlled by the circadian oscillator. The arrhythmic transcription pattern in *ccgs* was also not synchronized with *frq*, *wc-1*, nor *wc-2*. These data show that the circadian clock was suppressed and arrhythmic under DD conditions which negatively influenced downstream gene expression.

### Circadian gene transcription at steady state with periodic light exposure (LD)

**Figure 2:**
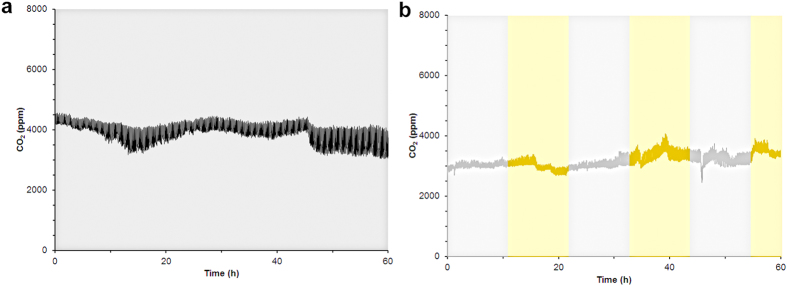
Representative real-time CO_2_ measurements from CSTR during gene transcription measurements under (a) DD or (b) LD cycles.

The periodicity of circadian gene regulation can be entrained by exposing a culture to light^35^. The data from DD experiments indicate that the transcription of circadian regulatory genes was low and arrhythmic in *N. crassa* when maintained in a steady metabolic state. A second set of experiments were performed with periodic (11 hr) light-dark cycles to determine if circadian gene expression can be entrained to light exposure under the same culture conditions. Samples for mRNA analysis were collected during steady state which was determined by the CO_2_ concentration monitored at the gas output from the vessel (Fig. 2b). No significant increase in CO_2_ output was observed during the light exposure periods which was an indication that light did not affect the metabolic state of the cells.

Three of the core circadian clock genes (*frq*, *wc-1,* and *vvd*) are known to respond to light stimuli and act as positive regulatory genes to synchronize gene expression^12^. The expression levels of *frq*, *wc-1*, and *vvd* increased immediately as a response to light exposure (Fig. 5a,b,d). However, a general trend of repression was observed among these genes during the remainder of the light exposure. As with the DD experiments, the presence of a rhythm was confirmed by time dependent statistical analysis (Supplementary Fig. S5-7). These results indicate that the transcription of these circadian genes were responsive to a light stimuli but suggest that the circadian clock might not be entrained and was operating independently of the light stimulation. In addition, the expression of *wc-2* was confirmed to be non-responsive to light exposure under these culture conditions (Fig. 5c), which is consist with *wc-2* being constitutively expressed^28^.

The expression of *ccgs* are affected by light exposure as well as controlled by the circadian clock^35^,^36^. To date, separating regulation of *ccgs* by the circadian clock and light sensitivity has not been shown. Specifically, *ccg-4* and *ccg-14* expression have been shown to be light sensitive as well as to be controlled by the circadian clock^36^. In our experiments, the expression of all *ccgs* responded to light exposure (Fig. 5e–g), indicating that the expression of these genes was up-regulated by the light stimulus without an operative circadian oscillator.

## Discussion

**Figure 3:**
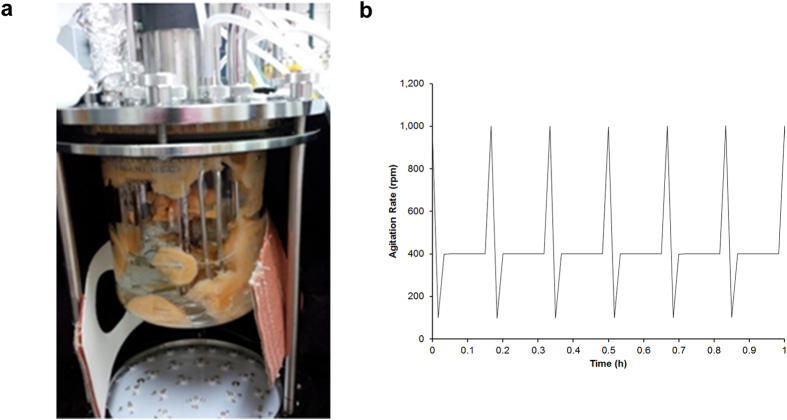
(a) Image from CSTR culture vessel containing *N. crassa* with Junlon (polyacrylic acid, MW ~100,000) but without dynamic agitation. (b) Dynamic Agitation program output. The propeller revolution rate was monitored (rpm) over time, cycling between 400 rpm (8 minutes), 1000 rpm (1 minute), and 100 rpm (1 minute) for the entire duration of the experiments. Cycling in a 1-hr period is shown to better visualize the program dynamics.

It is well-established that circadian gene oscillations are present and entrainable in racetube and batch culture assays using *N. crassa*^10^,^21^,^37^. The successful growth of *N. crassa* in an all-dark (DD) continuous culture reactor was also reported by Crosthwaite and coworkers in 2007 with no data presented for LD entrainment^26^. During their studies, periodic circadian oscillations (period: 20.2 ± 0.8 h) in *frq* transcription were observed in complete darkness over 96 hrs using glucose as the sole carbon source which is not consistent with the arrhythmic expression patterns observed during our experiments. Unfortunately, replication of their experimental conditions (including the use of Junlon as a fungal mat dispersing agent) resulted in large fungal mats (Fig. 3a) forming within the culture vessel when operated at a constant speed. These mats ultimately produced clogging issues in the waste removal lines and fouling of the dissolved oxygen probe and alternative methods needed to be employed.

The three significant differences between the experimental setup and results generated for this manuscript compared to the results presented by Crosthwaite and coworkers was the use of dynamic agitation to disperse the cellular material through the experiment, a significantly slower dilution rate (60.3 ± 0.7 mL/h vs. 0.074 ± 0.004 mL/h) to maintain steady state, and choice of reference genes (*btl* and *vma2*)^32^. The amount of CO_2_ generated remained steady between 3500–4000 ppm for both LD and DD experiments and confirmed that a steady metabolic state was maintained during these experiments (Fig. 2). There was also no change in CO_2_ production upon light exposure suggesting that the cellular activity was not effected in LD experiments. However, the exact reason that an 800 fold faster dilution rate resulted in a doubling of the amount of CO_2_/L (total gas) could not be determined since the overall gas flow rate was not reported in the previous work^26^. Interestingly, our higher dilution rate is consistent with a better dispersed biomass within the CSTRs.

To account for changes in multi-nucleated cellular states and to validate that RT-qPCR data was determining transcriptional changes and not changes in gene copy numbers our group has published data involving several reference genes using samples generated from these CSTRs^32^. Plotting both the RNA: DNA ratio and C_T_ values for four genes under DD and LD conditions over time indicated that C_T_ changes were due to fluctuations in transcript copy number. Additionally, a strong negative correlation was found to exist between the total RNA:DNA ratio and C_T_ values, demonstrating that C_T_ changes served as a reliable reflection of transcription and not gene copy number fluctuations. Thus, the amount of bulk cellular material used for the transcription data did not fluctuate during these experiments.

Collectively, these data demonstrate that circadian gene oscillations in the DD CSTR cultures were suppressed and arrhythmic immediately after the light was turned off but the circadian genes were up regulated in response to periodic light exposure. We propose two hypotheses for these results. First, the absence of periodic circadian gene transcription in CSTR cultures was due to dynamic agitation to disperse fungal mats while maintaining continuous culture conditions. The constant shearing of cellular components could disrupt cell-to-cell signaling which is preserved within the complex cellular mats that are left undisturbed in traditional assays^30^ and potentially present in the previous published bioreactor experiment^26^. This hypothesis would account for observing periodic oscillations in *frq* with batch culture experiments as these experiments contain potentially large fungal mats. The loss of extracellular communication by the dispersal of these mats could result in asynchrony among the individual cellular circadian clocks and disrupt rhythmic gene expression. Even though the circadian rhythms might be disrupted due to a lack of cell-to-cell communications, there was still a transcriptional response to light. Additionally, this light-responsive change influenced downstream *ccgs,* suggesting that light exposure could be used in this system to manipulate gene expression without an operative circadian clock which would circumvent negative feedback loops in other potential regulatory pathways. The ability of light to influence circadian gene expression in the absence of a functional circadian clock has previously been observed in *frq* mutant strains^38^,^39^.

If dynamic agitation was not the cause of the circadian clock disruption, then the second hypothesis is that the continuous cultivation (i.e., tightly controlled environmental conditions, consistent supply of fresh medium, and efflux of cellular material and byproducts) disrupts circadian gene regulation. Continuous cultivation would minimize environmental stressors (waste and/or lack of nutrients) that would be present during established assay conditions (i.e., racetubes and/or batch submerged cultures). These could also act as signals for cellular metabolism and reinforce circadian gene regulation in response to changing culture environments. Whereas, controlled culture conditions allow for cellular growth without uncontrolled zeitgebers and disrupts the regulatory timing of the organism.

One set of control experiments were performed to address the impact of dynamic agitation on the periodicity of *frq* transcription in these reactors. In this experiment the culture environment was left uncontrolled (batch; no influx of medium, efflux of cell material, or pH control (Supplemental figure S8) and dynamic agitation was used to keep fungal mats from forming. If the culture environment was left uncontrolled as in batch culture experiments and dynamic agitation did not result in loss of circadian rhythmicity, *frq* transcription under DD conditions should show a significant and periodic increase.

However, there were several issues in processing the samples from the control batch experiments with dynamic agitation. First, the amount of RNA isolated from a sample was decreased significantly (46 ± 5 ng/μL) in the batch (control) experiment compared to continuous culture experiments (260 ± 3 ng/μL; Supplementary Table S2). The reason for this decline in isolated RNA concentration (utilizing the same extraction method) has not been identified. However, one might conclude that the culture was starving due to the lack of replenishment of nutrients (i.e. continuous culture) and/or the cells were not transcriptionally active and thus the lysis of cells led to the decrease in RNA extracted. The unhealthy state of these cultures after 12 hrs was also reflected in the CO_2_ output from the reactors during the sampling period (data not shown) which decreased to 10% of what was observed during continuous culture experiments (Fig. 2a).

The second issue in processing the samples from this batch control experiment was instability of the reference genes validated from the continuous culture experiments used for RT-qPCR. In the DD and LD experiments presented in this manuscript, a detailed study had previously been published which addressed the issue of reference gene stability under the continuous culture conditions used for these experiments^32^. In that publication, *btl* and *vma2* were identified as stable reference genes for cultivating *N. crassa* in continuous culture bioreactors with dynamic agitation. Unfortunately, *vma2* and *btl* were not stable under control experimental conditions (Supplementary Fig. S9a and Tables S2 and S3). Therefore, *frq* transcript levels were only analyzed during the first 12hrs of the control experiment (Supplementary Fig. S9b) where *btl* and *vma2* were stable.

**Figure 4:**
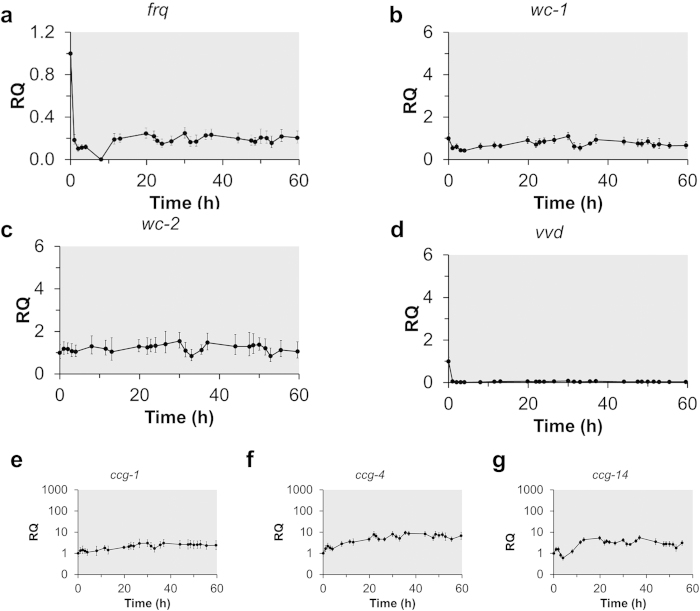
Gene expression analysis of *N. crassa* grown in a CSTR with continuous darkness (DD). *N. crassa* was cultured in Vogel’s medium containing 1% (w/v) glucose and 0.08% (v/v) polyacrylic acid (MW ~100,000) at 25 °C. Reactors were exposed to light for 24hrs following inoculation then maintained in constant darkness for the remainder of the experiment for data collection. The shaded region corresponds to the dark period. Gene expression of (**a**) frequency (*frq*), (**b**) white collar-1 (*wc*-1), (**c**) white collar-2 (*wc*-2), (**d**) vivid (*vvd*), and clock-controlled genes (**e**) *ccg-1*, (**f**) *ccg-4*, and (**g**) *ccg-14* were measured over time and were analyzed using the ΔΔC_T_ method with *btl* and *vma2* as reference genes. Shown are the data collected from one of three biological replicates, and error bars represent SD between triplicate samples within one biological replicate.

The magnitude and change in the level of *frq* transcription during this DD batch control experiment with dynamic agitation from the initial time point over 12 hrs is greater than at any point of the DD CSTR and is indicative of *frq* regulation in the dark using these batch conditions (Fig. 4). However, considering the compromised state of the cells based on CO_2_ output, unstable environmental conditions, and unstable housekeeping genes, we could not collect enough time points to confirm if this was an actual rhythm in *frq* transcription. The slight increase in *frq* regulation over 12 hrs suggests that dynamic agitation was not solely disrupting a rhythm in circadian gene transcription during the DD CSTR experiments.

Controlling gene expression with light and confounding cell-to-cell signaling is extremely advantageous from a biotechnology perspective. The CSTR system described here provides a method to define gene transcription with an external cue (i.e. light) without self-regulation through a circadian clock. The CSTR culture environment is significantly different from conventional experiments and “disorients” the natural timing of *N. crassa* which allows for self-regulatory networks to be circumvented. Thus, these data bring light to the potential importance of uncontrolled zeitgebers which are unaccounted for in accepted liquid submerged culture and solid substrate experiments used to study circadian rhythms in *N. crassa*.

## Methods

### Cell Maintenance and Batch Growth Conditions

A *Neurospora crassa* strain housing *ras-1*^*bd*^*; frq-luciferase* (C. Hong, University of Cincinnati) was used in all experiments. Cells were maintained on sucrose slants at −20 °C. Frozen cells were inoculated onto slants (2% Vogel’s 50x salts, 0.01% trace elements solution, 0.005% biotin, 1.5% sucrose, and 1.5% agar) and incubated at 30 °C for 2 – 3 days in the dark until orange conidia were observed. Conidia were isolated from slants using standard methods and inoculated into 50mL of fresh Vogel’s medium (2% Vogel’s 50x salts, 0.01% trace elements solution, 0.005% biotin, and 1.0% glucose) containing Junlon (Polyacrylic acid, MW 100,000, 0.2%, Sigma-Aldrich) and Antifoam 204 (0.008%, Sigma-Aldrich). After ~24 hrs of growth in light the 50 mL culture was inoculated into a 1.3L reactor vessel (New Brunswick BioFlow®/CelliGen® 115) with a final volume of 675 mL.

Racetube assays were performed consistent with procedures reported by other groups^28^. Briefly, conidia were inoculated onto agar medium (2% Vogel’s 50x salts, 0.01% trace elements solution, 0.005% biotin, 1.0% glucose, and 1.5% agar), exposed to light for 24hrs, then incubated in the dark for 5 days. Every 24 hrs the growth front was marked and the period of the banding pattern was determined as described visually using quantitative markings and alignment.

### CSTR Set-Up and Growth Conditions

CSTRs were operated and maintained according to protocols provided by New Brunswick, Inc. To minimize light exposure, the CSTRs were covered tightly with a custom three layer light exclusion shroud. The BioCommand Software (New Brunswick, Batch Control Plus Version B) was used to manage the CSTR controllers and collect data. The pH (5.5) and dissolved oxygen (DO,10%) were monitored throughout these experiments (Supplementary Fig. S2). The DO and pH probes were calibrated according to the manufacturer’s specifications provided by Mettler Toledo. Temperature was monitored using a temperature probe, and was controlled through an external heating blanket and internal cooling finger. A constant temperature of 25 °C was maintained throughout these experiments. An inline calibrated CO_2_ meter (Vernier) was placed within the vent gas output for the bioreactor and data was recorded using LoggerLite Software (Version 1.6.1). The gas flow rate was 1.0 L/min which was computer balanced between N_2_ and O_2_ inputs.

The dynamic agitation program cycled as follows: 400 rpm for 8 minutes, 1000 rpm for 1 minute, and 100 rpm for 1 minute. After inoculation the culture was grown in batch and exposed to light (3800 lumens, Tesler #W2233). The continuous influx of new medium and removal of material began ~22 hrs after the batch culture was inoculated. The culture was diluted at a rate of 1.0 mL/min which was determined to maintain a continuous culture and steady state in the vessel. A conductive level probe (New Brunswick) was used to control the flow of material from the culture vessel. The culture volume remained constant by syncing one of the control pumps to the triggering of liquid conductivity probe (New Brunswick). The previously described programs were designed in the BioCommand Software. At 11 hrs after the cells reached steady state growth the light was shut off and either remained off (DD), or cycled between on and off (L/D) with 11-hr periods.

For control batch DD experiments, the culture was inoculated and grown until steady-state was reached (as described above). For these experiments there was no continuous culture but dynamic agitation, when the light was shut off the continuous culture was also halted (influx/efflux) as well as pH control. The DO (set to 10%) and dynamic agitation program remained active throughout the sampling period.

### CSTR Sampling Conditions

**Figure 5:**
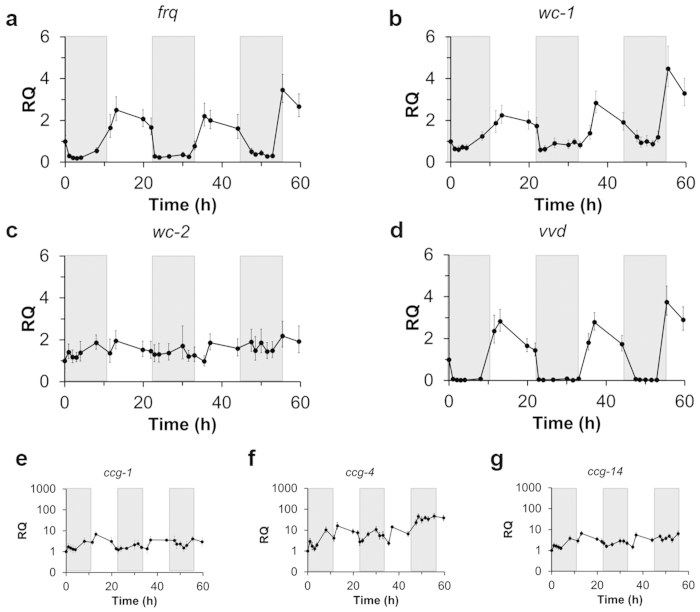
Gene expression analysis of *N. crassa*grown in a CSTR with periodic light/dark cycles. *N. crassa* was cultured in Vogel’s medium containing 1% (w/v) glucose and 0.08% (v/v) polyacrylic acid (MW ~100,000) at 25 °C. Reactors were exposed to light for 24 hrs following inoculation then exposed to cycles of darkness (11 hrs) then light (11 hrs) for the duration of data collection. The shaded region corresponds to dark periods. Gene expression of (**a**) frequency (*frq*), (**b**) white collar-1 (*wc*-1), (**c**) white collar-2 (*wc*-2), (**d**) vivid (*vvd*), and clock-controlled genes (**e**) *ccg-1*, (**f**) *ccg-4*, and (**g**) *ccg-14* were measured over time and were analyzed using the ΔΔC_T_ method with *btl* and *vma2* as reference genes. Shown are the data collected from one of three biological replicates, and error bars represent SD between triplicate samples within one biological experiment.

Samples were collected from CSTRs at ~2-4 hr intervals for 60+ hrs. Red darkroom safelights were used during sampling, regardless of the light conditions in the reactor. At each time point ~8 mL were collected from each reactor with 6 mL samples flash frozen in liquid nitrogen (lN_2_) immediately in 1 mL aliquots and stored at -80 °C until RNA extraction. Expression data from DD and LD CSTR experiments were generated from three biological replicates with the representative data from a single replicate presented in Figs. 4,5. The complete data sets from gene transcription of *frq* with SAS analysis for each individual experiment are provided in the supplementary information.

### Cell Lysis for Total RNA Extraction

Frozen 1 ml aliquots were thawed on ice and transferred to ice cold 2 ml Eppendorf tubes containing a 5 mm stainless steel bead (one bead per tube; Qiagen, Valencia, CA). Samples were centrifuged at room temperature for 1.5 min at 20,000 x g. The supernatant removed and the pellet was suspended in 100 μl of Buffer RLC from the Plant Mini Kit(Qiagen). The suspension was then subject to 4 freeze/thaw cycles which consisted of flash-freezing in liquid N_2_, thawing for 1 min at room temperature and incubating 5 min on ice. The suspension was vortexed for five seconds prior to the next freezing cycle. Cells were further disrupted using a TissueLyser LT (Qiagen). Samples were loaded into the ice cold adapter and processed for 2 min at 50 Hz, and then incubated on ice for 2 min. The homogenization/ice incubation process was repeated three times. 350 μl of buffer RLC was then added to the lysate, vortexed for five seconds, centrifuged briefly (94 x g, 5 sec, room temperature), and transferred to a sterile 2 ml Eppendorf tube.

### RNA Extraction

Total RNA was extracted using the RNesay Plant Mini Kit on the QIAcube automated system. Extraction was performed following the manufacturer’s protocol, with slight modifications: the DNase treatment was extended from 15 min to 1hr. Total RNA was eluted with 40 μl of RNase free water. Eluted fractions were placed on ice if being used immediately in the reverse transcription (RT) reaction or stored at -80 °C until use. Total RNA concentration and purity was assessed using the Nanodrop 2000 (Thermo Scientific, Wilmington, DE).

### Reverse Transcriptase Reaction

The reverse transcriptase reaction was performed using the High Capacity RNA-to-cDNA kit (Applied Biosystems®, Life Technologies, Grand Island, NY) with the following modifications: total RNA was diluted 1:5 in nuclease-free water, and 500 ng added to the reaction. Each reaction contained: 2 μL 20x enzyme mix, 2x RT buffer, a volume equivalent to 500 ng total RNA, and brought to a final volume of 40 μL with nuclease-free water. Negative RT Controls consisted of all components except the enzyme, in which case an equal volume of nuclease-free water was added. Reactions were incubated in a 2720 thermocycler (Applied Biosystems, Foster City, CA) under the following conditions: 37 °C for 60 min followed by heating to 95 °C for 5 min. Prior to use in RT-qPCR, samples were again diluted 1:5 in nuclease-free water. cDNA was stored at −20 °C for long term storage.

### RT-qPCR Reactions and Analysis

Quantitative PCR assays based on SYBR green chemistry were optimized for a subset of the genes (Table 1) known to be involved in clock control and light responsiveness. Primers were obtained from Life Technologies, and optimized over concentrations spanning 150-500 nM, with an annealing/extension temperature of 60 °C. Product specificity was verified via melt curve analysis (data not shown). Reactions were assembled with Qiagen’s QIAgility automated pipetting system. Reactions included 10 μL 2x Fast SYBR Green master mix (Life Technologies), 5 ng (2 μL) of cDNA, optimized primers concentrations (150 nM or 500 nM and brought to a final volume of 20 μL with nuclease-free water. The following protocol was used for all assays: an initial 20 s incubation at 95 °C, followed by 40 cycles of 95 °C for 1 s and 60 °C for 20 s, followed by a melt curve analysis of 95 °C for 15 s, 60 °C for 1 min, and 95 °C for 15 s to determine product specificity. All RT-qPCR reactions were performed in triplicate using Applied Biosystems MicroAmp Fast 384-well reaction plates sealed with MicroAmp optical adhesive film. No-template controls were also included in each amplification run to monitor for contamination. Reactions were recorded and analyzed using the Applied Biosystems ViiA7 Real-Time PCR System with 384-Well Block. Fold-change was calculated using the ViiA7 System software package with *btl* and *vma2* serving as the reference genes. Standard deviations of these data sets are presented in supplementary Table S1 of RQ values for the six biological replicates.

### Data analysis of periodic trends using SAS

Time dependent gene expression data (RQ) for *frq*, *wc*-1, and *wc*-2 from all of the DD and LD CSTR experiments were analyzed for time dependent periodic trends in expression. Data, in the form of average RQ values from three biological replicates, were input into SAS University Edition software (www.SAS.com). The SAS/ETS time series analysis methods were used which allows for determining periodic trends and cyclic patterns in time-dependent data series. The output chosen from this analysis included an autocorrelation (Supplementary Fig. S5-7 a, c) and a white noise test which was based on Ljung-Box Chi-Squared Statistics. The results from this analysis are shown in Supplementary Fig. S5-7 b,d with a logarithmic ordinate scale. Autocorrelation at lag k (ρ_k_) measures the correlation (similarity) of the time dependent variable at intervals = k. A high positive autocorrelation value indicates the values at intervals of k are likely to be similar, while a large negative value indicates that they are likely to be significantly different. The threshold for statistical significance for the autocorrelation values is determined by the standard error of the time series at lag k. A time series is a white noise time series (i.e. the variable is not dependent on time) if it has a constant mean (mean≠0), constant variance (σ^2^), and is uncorrelated (autocorrelation value does not exceed the threshold for significance) at all lag values. The Ljung-Box Chi-Squared Statistics displays the results of testing the null hypothesis that the time series is white noise at all lag intervals up to k. The null hypothesis is rejected (or the time series is rhythmic at lag k) at the 95% confidence level when the test statistic is less than 0.05 (blue line extends beyond the red line in Supplementary Fig. S5-7 b, d). Complete description of this method is provided online at www.SAS.com. The average RQ values from biological replicates were used as the input data for this analysis.

## Additional Information

**How to cite this article**: Cockrell, A. L. *et al.* Suppressing the *Neurospora crassa* circadian clock while maintaining light responsiveness in continuous stirred tank reactors. *Sci. Rep.* **5**, 10691; doi: 10.1038/srep10691 (2015).

## Supplementary Material

Supporting Information

## Figures and Tables

**Table 1:** Sequences of primers used for circadian and clock controlled genes.

**Gene Symbol**	**Sequence**	**Concentration (nM)**
*ccg-1* F	TCC CAC CTC CCC AAT ACC AT	500
ccg-1 R	CGT AGT TGG CAG CGT TCT TG	
*ccg-4* F	GTC ATG ACC GCC ATC CAG TC	500
*ccg-4* R	CAT CAC GCT TCC AGC AAA CC	
*ccg-14* F	GCT TGT GGG CTT TAG GGG AT	500
*ccg-14* R	TCC AAA CAG CTC CTT CCA CC	
*frq* F	GAA GGA CGA TTT GGC GTT CG	500
*frq* R	TGT CCA CCT CTT TTC CGG TG	
*vvd* F	GGA TAC AGC AAT GCG GAG GT	500
*vvd* R	CCG TCG GGT GAC TGA AGA AA	
*wc-1* F	TCG GCC CAT TGA CTA CAT CG	500
*wc-1* R	CCT ATG AGT CTG ACA GCG GC	
*wc-2* F	GAC ACT ATG CAA TGC CTG CG	500
*wc-2* R	ACC TCC GCC GTT ATT GTT GT	
*BTL* F	CCA CTT CTT CAT GGT CGG CT	150
*BTL* R	CTT GGG GTC GAA CAT CTG CT	
*VMA2* F	GTC GTC CAG GTC TTC GAG G	150
*VMA2* R	TGC CGG AAC CAT CAA AGA TAC G	

F = Forward and R = Reverse.
