# Supplementary material for: Suppressing the Neurospora crassa circadian clock while maintaining light responsiveness in continuous stirred tank reactors
Source: Sci Rep. 2015 Jun 2;5:10691. doi: 10.1038/srep10691 (PMC4451529; doi:10.1038/srep10691)
Supplement: Supporting Information [file srep10691-s1.pdf]

Supplemental Information for "**Suppressing the *Neurospora crassa* circadian clock while maintaining light responsiveness in continuous stirred tank reactors**"

Allison L. Cockrell[1], Russell K. Pirlo[1], David M. Babson[1†], Kathleen D. Cusick[1], Carissa M. Soto[2], Emily R. Petersen[3], Miah J. Davis[4], Christian I. Hong[5], Kwangwon Lee[6], Lisa A. Fitzgerald[1‡], and Justin C. Biffinger[1‡*]

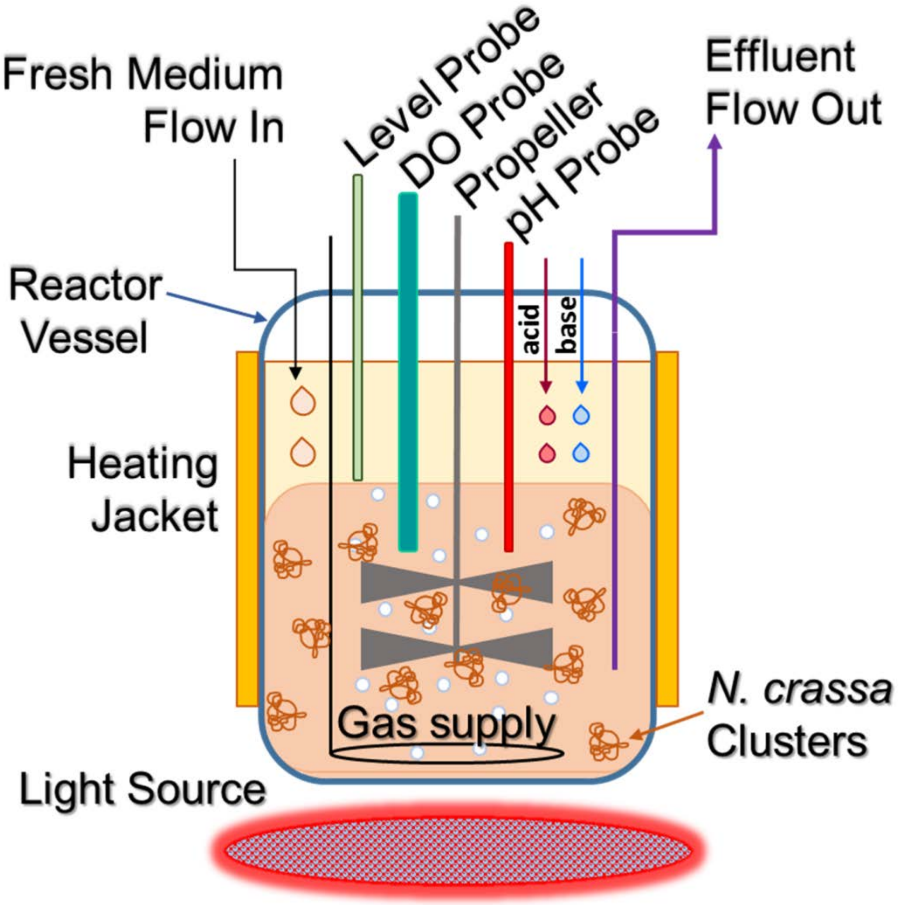

Figure S1. CSTR set-up. CSTR contained a stirring propeller to agitate the samples, a gas supply loop for O_2 and N_2 bubbling, and temperature regulation via an internal cooling finger and external heating jacket. The Dissolve O_2 (DO) probe and pH probe were interfaced with the BioCommand program (New Brunswick) to regulate the O_2 supply and acid/base addition. H_2SO_4 (1M) and NaOH (2M) were each supplied to the vessel through a peristaltic pump system which was controlled by the BioCommand program. Fresh medium flowed into the system at a rate of 1 mL/min and a level probe was used to maintain the fluid level in the reactor. The level probe was also interfaced with the BioCommand program and the fluid level was maintained by removing culture material. A light source was mounted under the reactor vessel to control light/dark cycles, and the entire reactor was shielded from any adventitious light in the room using a dark cloth lined with aluminum foil.

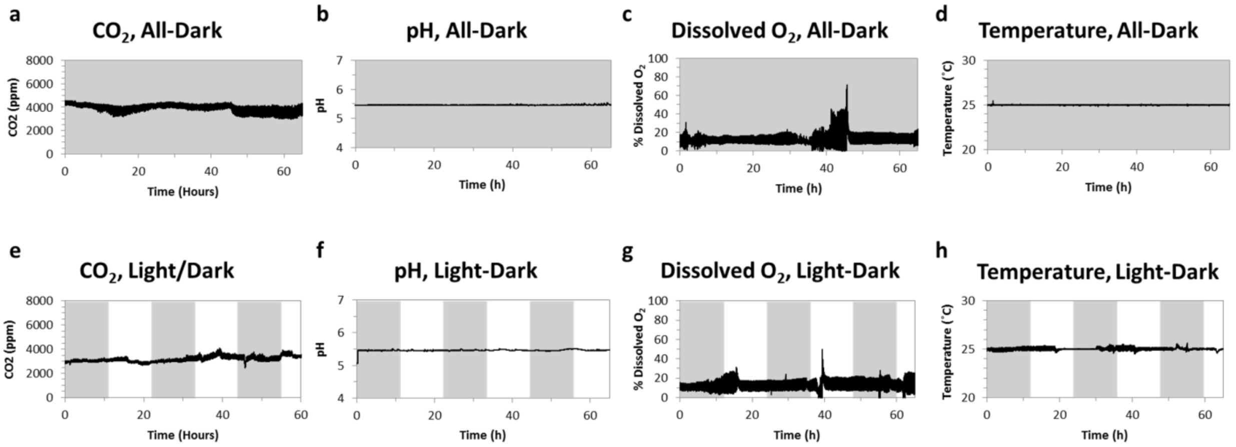

Figure S2. CSTR Output Data from DD and LD experiments. Levels of CO_2 output (a and e), pH (b and f), Dissolved Oxygen (c and g), and Temperature (d and h) were monitored in these experiments. a – d were collected during all-dark experiments, and e – h were collected during dark/light cycle experiments. The shaded areas represent points at which the lights were off.

Cell Staining and Imaging. *N. crassa* cells were stained according to Raju, 1982.[1] Briefly, fresh cells were fixed with 1% glutaraldehyde and frozen at -20˚C for ~1 – 2 days. The cells were thawed and washed with ddH_2O once, then suspended in DAPI solution (0.5 µg/ mL) and incubated for 10 minutes at room temperature. Cells were washed in ddH_2O twice, then suspended in 25% glycerol solution. ~1 hour before imaging, a drop of the cell suspension was placed on a clean glass slide and overlaid with a coverglass. Some samples were co-stained with Calcofluor white in image cellular bodies. In these samples, a drop of the cell suspension, 1M KOH (Sigma Aldrich), and 1M Calcofluor white (Fluka Analytics) were placed on a clean glass slide and overlaid with a coverglass. Images were collected on a fluorescent microscope (Nikon).

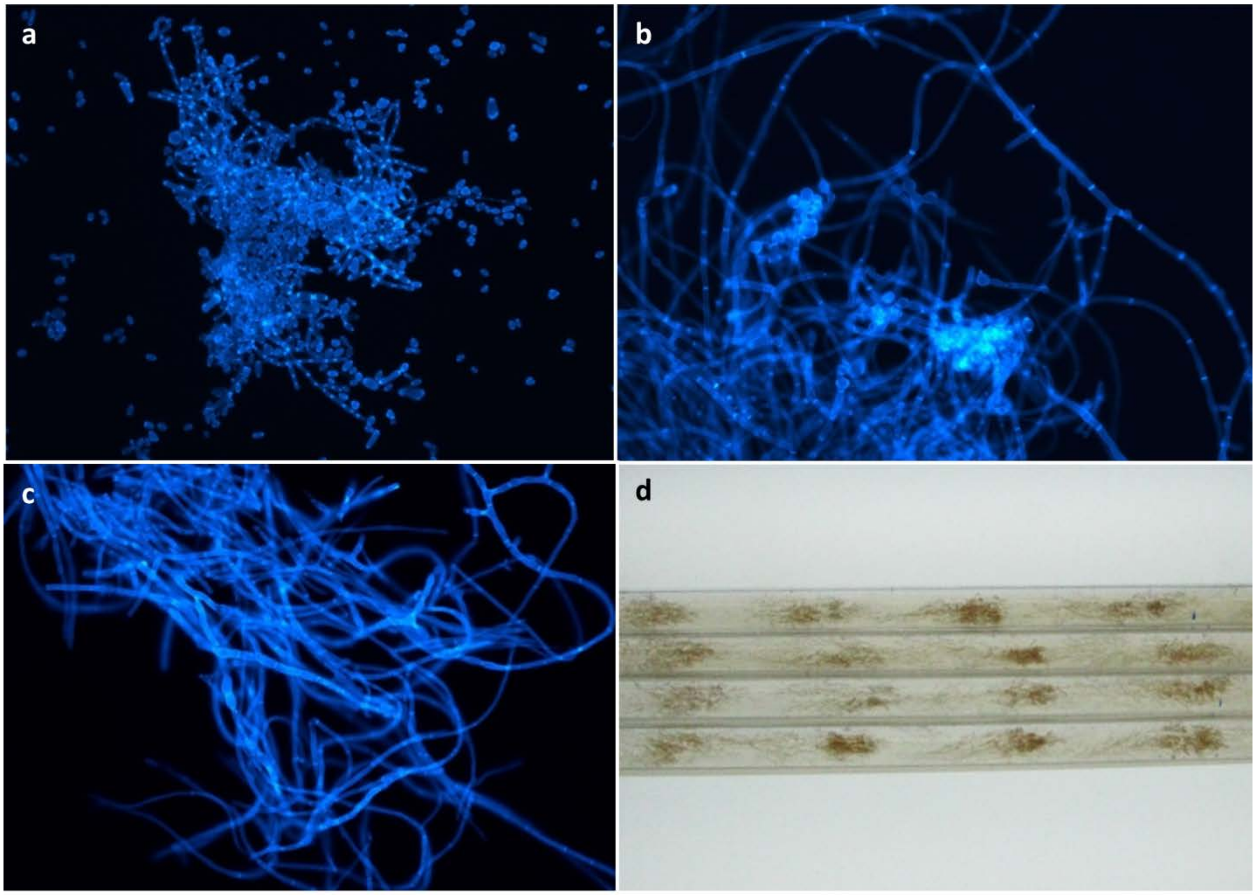

Figure S3. *Neurospora crassa* cells grown under different culture conditions. *N. crassa* conidia isolated from slants stained with Calcofluor white (a), cells grown in batch with Junlon (b), cells growing in CSTRs with Junlon and dynamic agitation (c), and cells grown in racetubes on agar containing 1% glucose (d) overhead photograph of time course racetube experiments containing *N. crassa* strain used for these experiments.

a

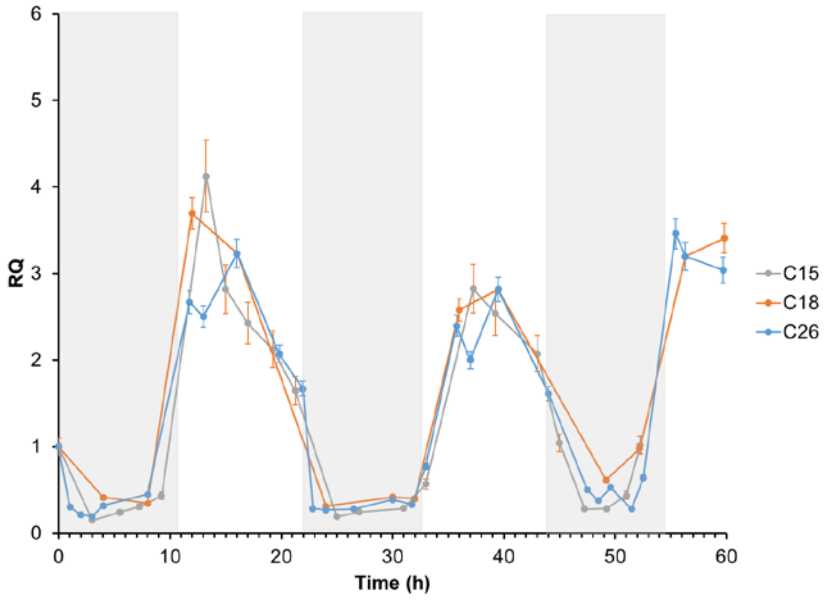

b

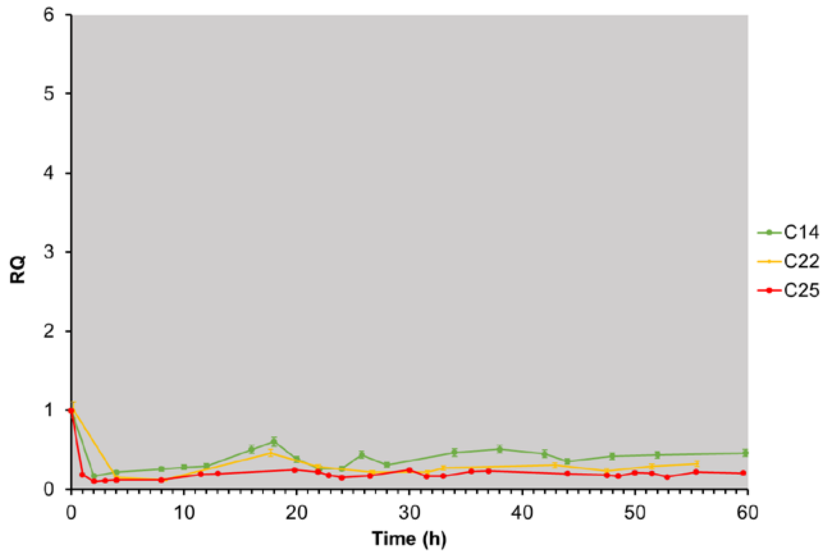

Figure S4. qRT-PCR *frq* transcription data for *N. crassa* grown in a CSTR with periodic (a) light/dark (LD) cycles or in (b) constant darkness (DD) over the experimental time period. CSTR #15 (C15), CSTR #18 (C18), and CSTR #26 (C26) are labels for individual replicate CSTRs operated with LD cycles. CSTR #14 (C14), CSTR #22 (C22), and CSTR #25 (C25) are labels for individual replicate CSTRs operated in constant darkness (DD). The shaded region corresponds to dark periods. Standard errors are shown in the representative colors from triplicate qRT-PCR reactions. RQ was normalized to the reference genes *btl* and *vma2*.

Table S1. Standard deviation between three biological replicates in LD and DD CSTR data sets using RQ values from RT-qPCR data for *frq* (in triplicate)

	LD	DD
Number of replicates	3	3
Standard Deviation RQ (light on)	0.15	n/a
Standard Deviation RQ (light off)	0.072	0.062
Standard Aggregate Deviation RQ (all data)	0.16	0.062

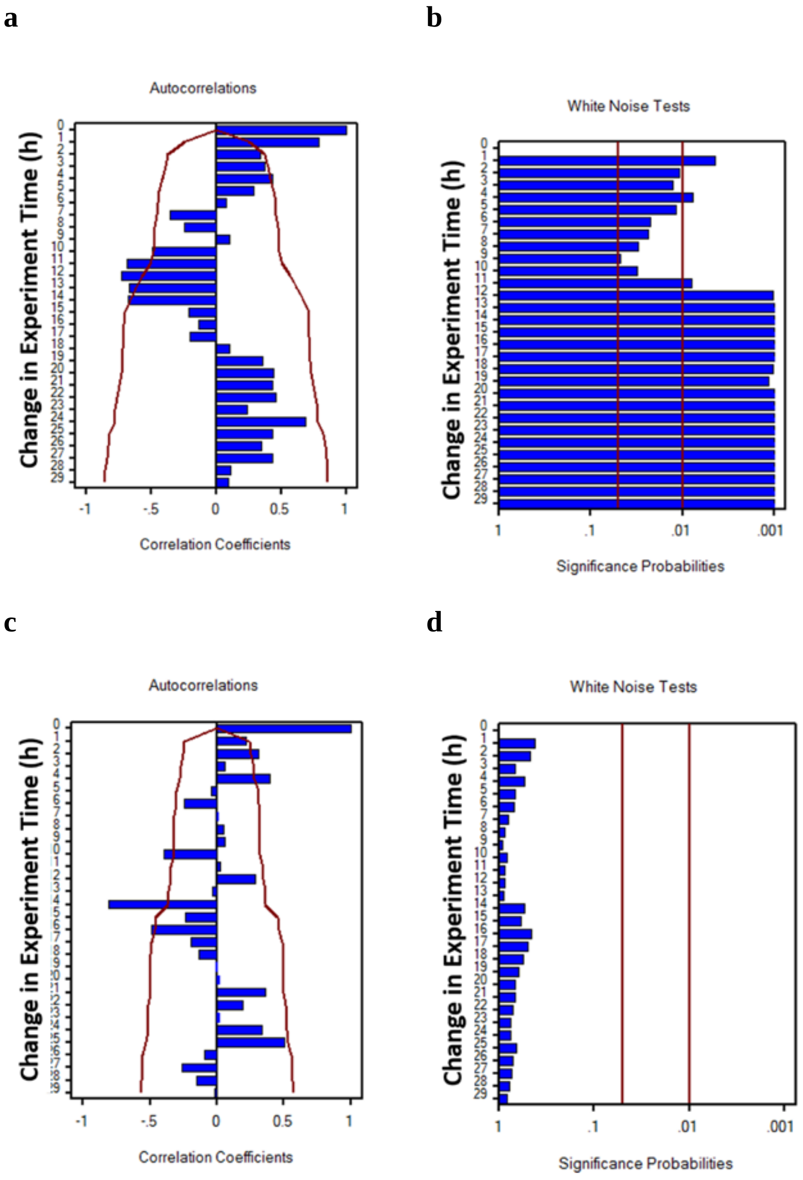

Figure S5. Correlation of *frq* gene expression in LD and DD CSTR experiments with time. Autocorrelation data output from SAS/ETS statistics package using time dependent gene expression data (RQ) from all CSTR replicates for *frq* expression from the (a) LD and (c) DD conditions with white noise probability output (b) and (d). All data were input in SAS University Edition software (www.SAS.com).

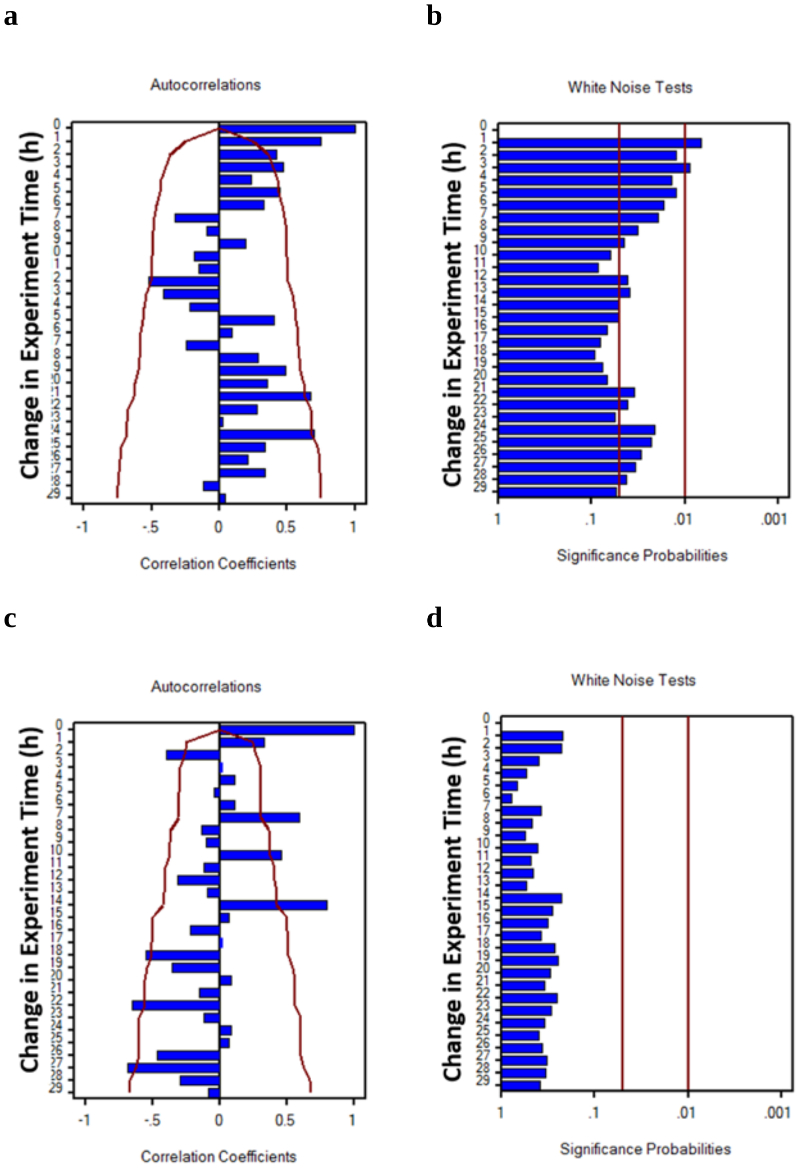

Figure S6. Correlation of *wc-1* gene expression in LD and DD CSTR experiments with time. Autocorrelation data output from SAS/ETS statistics package using time dependent gene expression data (RQ) from all CSTR replicates for *wc-1* expression from the (a) LD and (c) DD conditions with white noise probability output (b) and (d). All data were input in SAS University Edition software (www.SAS.com).

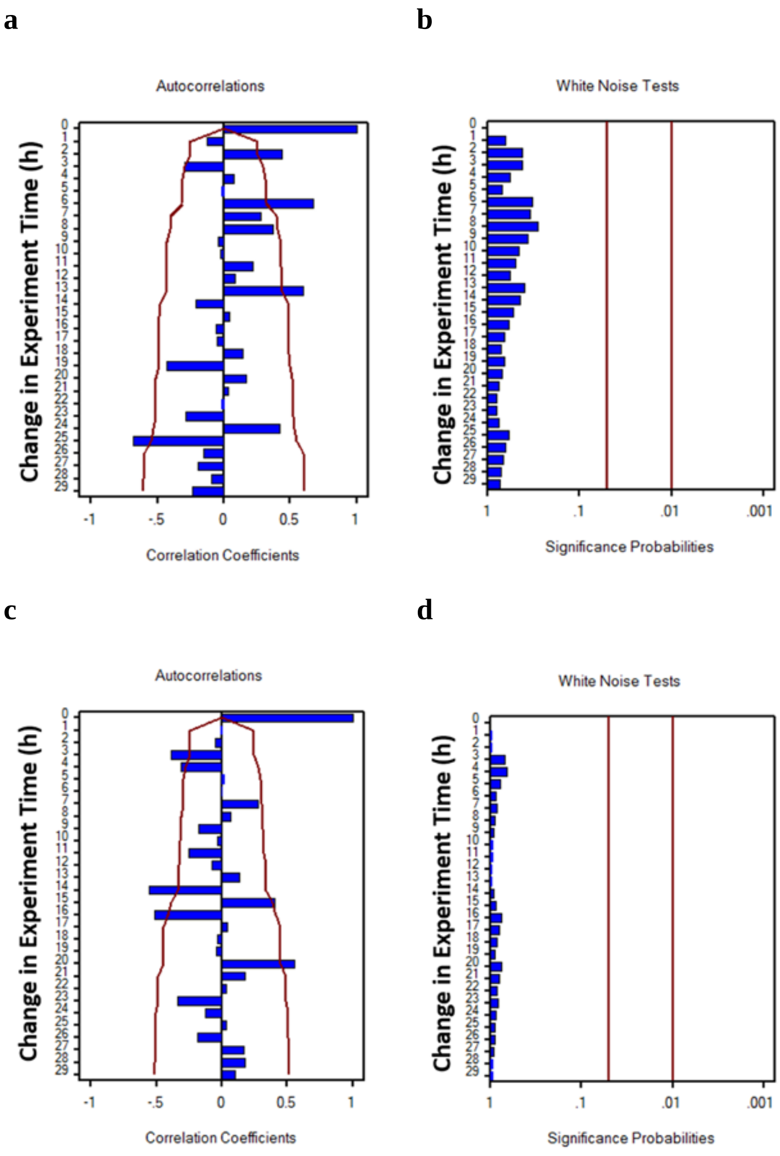

Figure S7. Correlation of *wc-2* gene expression in LD and DD CSTR experiments with time. Autocorrelation data output from SAS/ETS statistics package using time dependent gene expression data (RQ) from all CSTR replicates for *wc-2* expression from the (a) LD and (c) DD conditions with white noise probability output (b) and (d). All data were input in SAS University Edition software (www.SAS.com).

DD – Dynamic Agitation – Continuous Culture		DD – Dynamic Agitation – Batch (Control) Culture	
# of Samples	40	# of Samples	12
Average RNA Conc.	264.5	Average RNA Conc.	46.0
Median	228.8	Median	44.9
Maximum RNA Conc.	625.6	Maximum RNA Conc.	11.7
Minimum RNA Conc.	82.0	Minimum RNA Conc.	121.8

Table S2. Concentrations of extracted RNA in Continuous and Batch (Control) Culture Experiments. The average, median, minimum, and maximum concentrations of RNA extracted from the continuous culture experiment and batch culture experiment with dynamic agitation are presented in this table. Concentrations are reported as ng/μL.

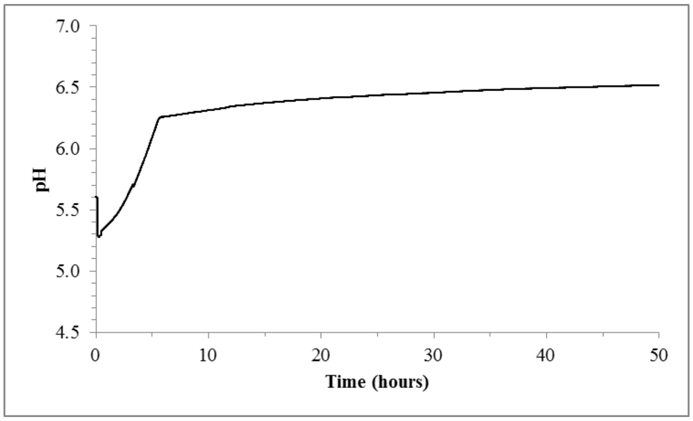

Figure S8. Change in acidity observed during batch (control) experiment during sampling period in DD

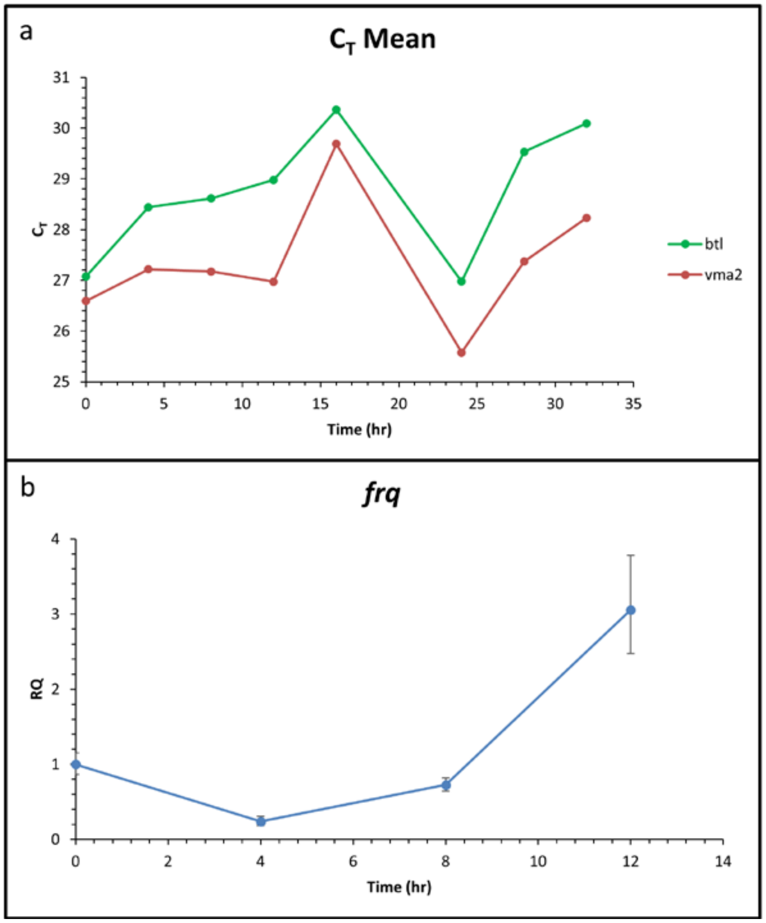

Figure S9. Gene transcription during batch culture with dynamic agitation. *N. crassa* was cultured in Vogel's medium containing 1% (v/v) glucose and 0.08% (v/v) polyacrylic acid (MW 100,000) at 25°C. Reactors were exposed to light for 24hrs following inoculation then maintained in constant darkness without continuous culture but with dynamic agitation for the remainder of the experiment for data collection. The top panel (**a**) shows C_T values of HKGs *btl* (green) and *vma2* (red) during 32 hours of cultivation. The bottom panel (**b**) shows gene expression of frequency (*frq*) measured during the first 12hrs of batch culture growth and was analyzed using the ΔΔCT method with *btl* and *vma2* as reference genes. Error bars represent SD between triplicate samples within one biological replicate.

	btl	*vma2*
SD, DD Continuous Culture	0.584	0.729
SD, DD Batch Culture	1.262	1.204
SD, DD Batch Culture (12hr)	0.833	0.286

Table S3. Standard deviations of HKG C_T values during continuous and batch (control) culture experiments. *N. crassa* was cultured in Vogel's medium containing 1% (v/v) glucose and 0.08% (v/v) polyacrylic acid (MW 100,000) at 25°C. Reactors were exposed to light for 24hrs following inoculation then maintained in constant darkness with or without continuous culture and with dynamic agitation for the remainder of the experiment. This table shows standard deviations between HKG C_T values during continuous and batch cultivation. Continuous cultures were analyzed over 60hr, and batch cultures were analyzed over 32hr. More information regarding HKGs in continuous bioreactor cultures is available in Ref. 40 (Cusick, et al., 2014, *PLoS One*).
